# Draft Genome Sequence of Lactobacillus crispatus CIP 104459, Isolated from a Vaginal Swab

**DOI:** 10.1128/MRA.01373-19

**Published:** 2020-01-09

**Authors:** Maximilien Clabaut, Amine M. Boukerb, Pierre-Jean Racine, Chantal Pichon, Coralie Kremser, Jean-Pierre Picot, Madina Karsybayeva, Gérard Redziniak, Sylvie Chevalier, Marc G. J. Feuilloley

**Affiliations:** a Laboratoire de Microbiologie Signaux et Microenvironnement (LMSM), EA4312, Université de Rouen Normandie, Normandie Université, Évreux, France; b Centre de Biophysique Moléculaire, CNRS UPR4301, Orléans, France; c GimoPharm, Longjumeau, France; d Seqens, Limoges, France; e Remedials Laboratoire, Paris, France; f Cosmetic Inventions, Antony, France; University of Maryland School of Medicine

## Abstract

We report the draft genome sequence of Lactobacillus crispatus CIP 104459, isolated from a human vaginal swab. This draft genome consists of 1,993,673 bp, with 36.8% G+C content and 2,024 predicted protein-encoding sequences.

## ANNOUNCEMENT

Lactobacilli are the predominant vaginal microbiota found in healthy women of reproductive age. Several studies have revealed that vaginal disorders and dysbiosis are closely related to lactobacillus decrease and pathogen increase (1). Lactobacillus crispatus is one of the dominant species in the normal vaginal environment and plays an important role in preventing infections such as bacterial vaginosis (BV) and in modulating inflammation (2). L. crispatus is a producer of lactic acid, hydrogen peroxide, and other antimicrobial compounds that inhibit the growth (3), hyphae, and biofilm formation of Candida albicans (4) and reduce the cytotoxicity of Gardnerella vaginalis (5) and the infectivity of Chlamydia trachomatis (6). L. crispatus seems to possess additional protective mechanisms against BV and contributes to the maintenance of the normal vaginal microbiota (2).

L. crispatus CIP 104459 was isolated on 1 January 1955 from a human vaginal swab in the maternity ward of La Croix-Rousse Hospital (Lyon, France). This strain was obtained from the Collection of Institut Pasteur (CRBIP—microorganism biobank catalogue) in Paris, France. The bacterial isolate was grown anaerobically onto de Man, Rogosa, and Sharpe (MRS) medium agar plates (ISO, VWR reference 84607.0500) overnight at 37°C under static conditions. The purified bacterial isolate was obtained by single-colony isolation, which was later maintained at −80°C using MRS medium supplemented with 20% glycerol.

In a first step, L. crispatus strain CIP 104459 was identified by total proteome analysis (data not shown). Subsequently, 16S rRNA gene sequencing was performed, and the blast algorithm allowed sequence comparison with other L. crispatus strains reported in the NCBI 16S RefSeq database. Since the 16S rRNA gene sequence of strain CIP 104459 displayed 100% sequence identity with those of L. crispatus strains V4, CO3MRSI1, and AB70 (GenBank accession numbers SRLG00000000, CP033426, and CP026503, respectively), CIP 104459 was identified as belonging to the species L. crispatus.

Genomic DNA was extracted from L. crispatus CIP 104459 grown in MRS broth for 24 h using a GeneJET genomic extraction kit, as per the manufacturer’s instructions (catalog number K0721; Thermo Scientific), after treatment for 60 min with a lysozyme-containing solution (20 mM Tris-HCl [pH 8.0], 2 mM EDTA, 1.2% Triton X-100, 20 mg/ml lysozyme). The genomic DNA was then sequenced on an Illumina MiSeq platform, as described by Clabaut et al. (7), using 250-bp paired-end (PE) read sequencing.

Default parameters were used for all software, unless otherwise noted. Sequencing resulted in 1,708,490 raw PE reads that were trimmed and quality filtered using Trimmomatic v0.36 (8) and then assembled into contigs using Unicycler v0.4.7 (9), with default parameters. This resulted in 428× coverage and 124 contigs, with a G+C content of 36.8%, an *N*_50_ value of 38,667 bp, an *L*_50_ value of 19, and a genome size of 1,993,673 bp, as calculated using QUAST v5.0.0 (10). Whole-genome *de novo* gene prediction was performed using Prokka v1.13.4 (11) This prediction resulted in 2,024 putative protein-encoding genes, 59 tRNA genes, and 3 rRNA genes.

### Data availability.

This whole-genome sequencing project has been deposited at DDBJ/ENA/GenBank under BioProject accession number PRJNA557339, and the corresponding assembly was deposited under accession number VOMA00000000. The version described in this paper is the first version, VOMA00000000.1. The raw data reads have been deposited in the Sequence Read Archive (SRA) database under accession number SRR9860122.
